# Ascorbic Acid Reduces Neurotransmission, Synaptic Plasticity, and Spontaneous Hippocampal Rhythms in In Vitro Slices

**DOI:** 10.3390/nu14030613

**Published:** 2022-01-30

**Authors:** Segewkal H. Heruye, Ted J. Warren, Joseph A. Kostansek IV, Samantha B. Draves, Stephanie A. Matthews, Peter J. West, Kristina A. Simeone, Timothy A. Simeone

**Affiliations:** 1 Department of Pharmacology & Neuroscience, School of Medicine, Creighton University, Omaha, NE 68174, USA; SegewkalHeruye@creighton.edu (S.H.H.); TedWarren1@creighton.edu (T.J.W.); JoeKostansek@creighton.edu (J.A.K.IV); samanthadraves@creighton.edu (S.B.D.); stephaniematthews@creighton.edu (S.A.M.); kristinasimeone@creighton.edu (K.A.S.); 2 Department of Pharmacology & Toxicology, College of Pharmacy, University of Utah, Salt Lake City, UT 84112, USA; peter.west@utah.edu

**Keywords:** high frequency oscillation, fEPSP, fiber volley, sharp wave, population spike, E-S coupling, LTP, T-type calcium channel

## Abstract

Ascorbic acid (AA; a.k.a. vitamin C) is well known for its cellular protection in environments of high oxidative stress. Even though physiological concentrations of AA in the brain are significant (0.2–10 mM), surprisingly little is known concerning the role of AA in synaptic neurotransmission under normal, non-disease state conditions. Here, we examined AA effects on neurotransmission, plasticity and spontaneous network activity (i.e., sharp waves and high frequency oscillations; SPW-HFOs), at the synapse between area 3 and 1 of the hippocampal cornu ammonis region (CA3 and CA1) using an extracellular multi-electrode array in in vitro mouse hippocampal slices. We found that AA decreased evoked field potentials (fEPSPs, IC_50_ = 0.64 mM) without affecting V_50_s or paired pulse facilitation indicating normal neurotransmitter release mechanisms. AA decreased presynaptic fiber volleys but did not change fiber volley-to-fEPSP coupling, suggesting reduced fEPSPs resulted from decreased fiber volleys. Inhibitory effects were also observed in CA1 stratum pyramidale where greater fEPSPs were required for population spikes in the presence of AA suggesting an impact on the intrinsic excitability of neurons. Other forms of synaptic plasticity and correlates of memory (i.e., short- and long-term potentiation) were also significantly reduced by AA as was the incidence of spontaneous SPW-HFOs. AA decreased SPW amplitude with a similar IC_50_ as fEPSPs (0.65 mM). Overall, these results indicate that under normal conditions AA significantly regulates neurotransmission, plasticity, and network activity by limiting excitability. Thus, AA may participate in refinement of signal processing and memory formation, as well as protecting against pathologic excitability.

## 1. Introduction

Ascorbic acid (AA), commonly known as Vitamin C, is among the vital water-soluble vitamins with non-enzymatic antioxidant properties. AA prevents the oxidation of macromolecules via inhibition of free radical chain reactions by scavenging reactive oxygen species (ROS) and reactive nitrogen species [1,2]. AA is involved in the regeneration of antioxidants such as α-tocopherol and glutathione and is also a co-factor in various enzymatic reactions [3,4]. Higher primates lack the functional enzyme essential for synthesis, thus requiring dietary supplementation to attain levels of AA necessary for physiological processes [5].

AA is differentially concentrated throughout the fluids and tissues of the body via sodium-vitamin C co-transporters (SVCT2) and glucose transporters (GLUTs). Coupling AA transport to sodium and glucose naturally gathers AA in areas of high metabolic demand where there is a greater rate of free radical formation during energy production and consumption. The brain requires over 20% of all oxygen metabolized in the body even though its mass is small relative to the whole [6]. Synaptic transmission is the primary consumer of the energy produced in the brain and high rates of neuronal activity form even more free radicals [7]. Whereas AA in plasma hovers around 40–60 µM, the concentration of AA in brain tissue ranges from 2 mM to 10 mM with the highest concentrations in the amygdala, hippocampus, and hypothalamus; with intracellular astrocytic concentrations being 1 mM, neuronal concentrations being10 mM, and extracellular concentrations ranging from 200 µM to 400 µM [4,8].

AA is neuroprotective in in vitro and in vivo models of neurological disease associated with marked oxidative stress such as stroke, Alzheimer’s, Parkinson’s, and Huntington’s [4,8,9]. The antioxidant properties of AA prevent cell death and preserve or improve impaired neurotransmission giving rise to the theory that the primary role of AA in the brain is to reduce free radicals and limit injury [4,10,11]. However, there is evidence that AA may impact neurotransmission more directly by involvement in the synthesis, release, and uptake of monoamines and catecholamines as well as allosterically modulating T-type Ca^2+^ channels, NMDA receptors, dopamine receptors and, 5-HT3 receptors and GABA receptors [4,10,12,13,14,15]. This predicts that even during normal conditions physiological AA should have an active role in neurotransmission. Surprisingly, there is a dearth of experimental investigation in this area. Here, we determined whether acute application of physiological concentrations of AA influence neurotransmission, synaptic plasticity and network oscillations. We examine these parameters at the synapse between area 3 and 1 of the hippocampal cornu ammonis region (CA3 and CA1) as this synapse is important for learning and memory.

## 2. Materials and Methods

### 2.1. Animals

Wildtype mice of C3HeB/FeJ strain were bred and maintained in the animal research facility of Creighton University School of Medicine. Mice were kept on a 12-h light/dark cycle and had *ad libitum* access to food and water. All experiments were approved by the Institutional Animal Care and Use Committee of Creighton University School of Medicine (Protocol No. 0875).

### 2.2. Chemicals

All chemicals were purchased from Sigma-Aldrich (St. Louis, MO, USA). A stock solution of 100 mM L-ascorbic acid (A92902) was prepared using double-distilled water and diluted with artificial cerebrospinal fluid to 0.2 mM, 0.4 mM, 1 mM and, 2 mM working solutions for perfusion.

### 2.3. Multi-Electrode Array Recording

**Figure 1:**
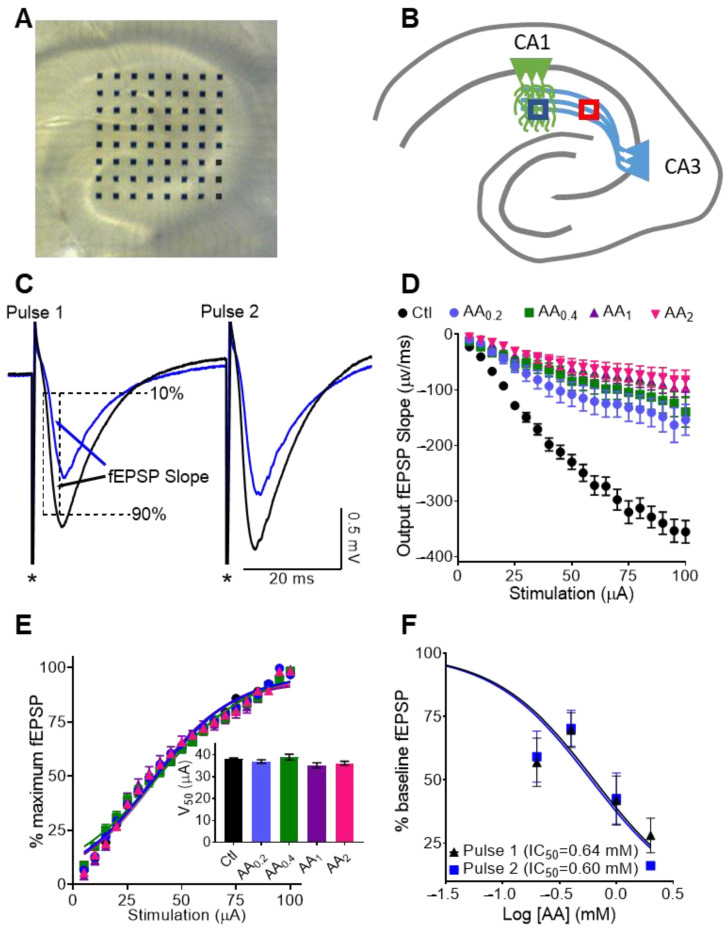
Physiological concentrations of AA reduce dendritic fEPSPs at CA3-CA1 synapses. (**A**) Brightfield photomicrograph of a hippocampal slice on a 64-electrode array. (**B**) Schematic of the stimulation electrode (pink) in the CA3 schaffer collaterals and the recording electrode in the CA1 stratum radiatum (blue). (**C**) Representative paired pulse traces before (black line) and after 0.2 mM AA application (blue line) illustrating the measurement of dendritic fEPSP slopes and inhibitory effect of AA. Asterisks demark stimulation artifacts. (**D**) Raw input/output (I/O) relationship between stimulus (µA) and the 10–90% slope of evoked fEPSPs. Output fEPSP slope was significantly reduced with all concentrations of AA. Statistical analyses were performed with Friedman test with Dunn’s multiple comparison post-hoc test. (*n* for Ctl = 16 mice, 16 slices, 89 electrodes; *n* for AA_0.2_ = 4 mice, 4 slices, 21 electrodes; *n* for AA_0.4_ = 4 mice, 4 slices, 29 electrodes; *n* for AA_1_ = 4 mice, 4 slices, 22 electrodes; *n* for AA_2_ = 4 mice, 4 slices, 17 electrodes). (**E**) Normalized fEPSP I/O fit with Boltzmann sigmoidal equation to calculate the V_50_ (inset). There was no difference in V_50_ between different concentrations of AA (*p* > 0.05; Welch’s ANOVA with Dunnett’s T3 multiple comparisons post-hoc test). (**F**) Concentration response curve. Normalized fEPSP slopes evoked with initial pulse (1) and second pulse (2) with a 40 ms interstimulus interval. The IC_50_ was calculated for both slopes from the curve fit by a Hill equation.

Postnatal day (P) 38–P45 mice were anesthetized, decapitated, brains removed, and horizontal sections (400 μm) of ventral hippocampal-entorhinal cortex were sliced on a vibratome (Leica VT1200, Leica Microsystems Inc., Bannockburn, IL, USA) positioned over a multi-microelectrode array on a MED64 probe (Figure 1A) as we have previously described [16,17,18,19]. One slice per animal was used. Spontaneous and evoked extracellular field recordings were acquired with Med64 Mobius software v3.02 (Alpha Med Scientific Inc., Osaka, Japan). For each slice, the experimental protocol was as follows: Initially, a single biphasic pulse (35 μA, once per minute) stimulated the Schaffer collateral pathway until responses in the CA1 stratum radiatum and stratum pyramidale stabilized for at least 15 min. An input/output (I/O) curve was then generated to determine the intensity of stimulus that elicited 40–60% of the maximum response at. Slopes (10–90%) of field excitatory post-synaptic potential (fEPSP) were used to quantify responses to limit contamination from population spikes and other sources of current within the slice, whereas waveform area was measured to quantify population spikes in the stratum pyramidale. Evoked potentials were then generated by paired pulses (40 ms inter-stimuli interval) every minute. The degree of facilitation was determined by calculating the paired-pulse ratio (PPR), i.e., dividing the second response by the first response. After steady control baseline responses were established for 15 min, spontaneous activity was recorded for five minutes, after which paired pulse stimulations resumed. Slices were then perfused with different concentrations of AA (one concentration per slice) until the effect plateaued (15–20 min). Another I/O curve was generated to determine AA effects on synaptic strength and excitability, and another five minutes of spontaneous activity was recorded. This was followed by LTP induction and additional recording of paired pulse stimulation for 60 min. LTP was induced with a theta burst stimulation (TBS) protocol consisting of eight trains (40 ms duration) of eight pulses at 200 Hz with 2 s intervals between trains and at a stimulus strength of 40–60%) as described by Sheridan et al. [20]. For measurements of control LTP, the same protocol was performed on untreated slices. Thus, slices from 20 mice (4 mice for each AA concentration and control LTP) were used to determine AA effects on neurotransmission, plasticity and network activity.

Binary Med64 data of the five-minute spontaneous activity recordings were imported to Spike2 (v7.19) software (Cambridge Electronic Design, Cambridge, England) and rescaled. Raw spontaneous activity recordings were subjected to notch filters (60, 180 and 300 Hz) followed by a 50 Hz low-pass second order filter (−3 dB point = 70 Hz), and down-sampled to 2.5 kHz to identify DC shifts of the SPWs. SPW detection threshold was 3 times the amplitude of the peak-trough noise and SPW characteristics (interval, duration, and amplitude) were determined. To visualize HFOs, filtered spontaneous activity recordings were band pass filtered 100–600 Hz (−3 dB points = 80 Hz and 619 Hz; 1319 filter coefficients). Threshold of HFO detection was 4 times the standard deviation of the root mean square of the noise calculated using a 0.1 s sliding window. HFO characteristics (interval, duration, and internal frequency) were determined with burst analyses [16,18,19]. Burst events were accepted if they consisted of at least three consecutive cycle troughs with inter-trough intervals of between 10 ms and 30 ms.

### 2.4. Statistical Analysis

All data were presented as mean ± SEM. A Boltzmann sigmoidal curve was used to fit IO data normalized to the maximum output, to determine the stimulation intensity that resulted in 50% of the response (V_50_). Nonlinear regression was used to construct a concentration response curve of normalized fEPSP slope to log AA concentration and the concentration that resulted in 50% inhibition (IC_50_) was calculated. Linear regression was used to obtain ratios of fiber volley-fEPSP (FV-E) coupling, whereas non-linear regression was used for fEPSP-population spike (E-S) coupling. A least square method of nonlinear regression was used to fit the average LTP. Span and plateau of the monoexponential fit were used to calculate the amplitude of potentiation for short-term potentiation (STP) and long-term potentiation (LTP), respectively. Kolmogorov-Smirnov test and Bartlett’s test were used to test normality and homoscedasticity of residuals respectively. Friedman test with Dunn’s multiple comparison post-hoc test, Wilcoxon matched-pairs signed rank test, and Welch’s ANOVA with Dunnett’s T3 multiple comparisons post-hoc test were used if there were violation of assumptions. Paired *t*-tests were used to compare regression slopes for FV-E and E-S coupling. All statistical analyses were conducted with Prism 8 software (Graphpad Software, Inc., La Jolla, CA, USA) and *p*-values < 0.05 were considered statistically significant.

## 3. Results

### 3.1. Physiological Concentrations of AA Reduce Dendritic fEPSPs at CA3-CA1 Synapses

Hippocampal slices were placed over the multi-electrode array (Figure 1A). To determine the effect of physiological concentrations of AA on synaptic transmission and plasticity, we stimulated CA3 Schaffer Collaterals with paired pulses and recorded field excitatory postsynaptic potentials (fEPSPs) in the CA1 stratum radiatum dendritic region (Figure 1B). AA effects were noticeable within 3 min of the start of perfusion and plateaued in 15–20 min (Figure 1C). Input (stimulation intensity)—output (fEPSPs) experiments revealed that AA significantly inhibited fEPSPs in a concentration dependent manner (57%, 61%, 73%, and 77% inhibition by 0.2, 0.4, 1 and 2 mM, respectively, at 100 μA stimulation) (Figure 1D) but did not affect the stimulation intensity required to elicit a half-maximal response (V_50_; Ctl = 38.0 ± 0.5 µA; 0.2 mM AA = 36.9 ± 0.8 µA; 0.4 mM AA = 38.9 ± 1.3 µA; 1 mM AA = 35.2 ± 1.3 µA; 2 mM AA = 36.1 ± 0.9 µA; one way ANOVA; *p* > 0.05) (Figure 1E). These data indicate that AA reduces synaptic strength, but not synaptic excitability.

### 3.2. AA Reduces Paired Pulse Slopes 1 and 2 Equally, thus Presynaptic Neurotransmitter Release Probablity Is Not Affected

We next determined whether presynaptic mechanisms contributed to the reduction of dendritic fEPSPs. First, we examined the effect of AA on paired pulse facilitation. Paired pulse facilitation is a form of short-term plasticity that lasts less than one second and is inversely related to presynaptic neurotransmitter release probabilities. AA inhibited both dendritic fEPSP slopes 1 and 2 to similar degrees. The AA concentration that inhibited 50% of the fEPSP (IC_50_) at 40–60% stimulation intensity exerted a similar inhibition on the paired pulse fEPSPs (Pulse 1 = 0.64 ± 0.13 mM; Pulse 2 = 0.6 ± 0.11 mM) (Figure 1F). These data suggest that AA attenuates paired pulse dendritic fEPSPs.

Paired pulse facilitation is a form of short-term plasticity that lasts less than one second and is inversely related to presynaptic neurotransmitter release probabilities. The paired pulse ratio (Slope2/Slope1) under control conditions was 142 ± 3.2%. AA inhibited the responses to Pulse 1 and 2 to similar degrees, thus the paired pulse ratio did not differ from control (data not shown). These data suggest that AA does not affect presynaptic neurotransmitter release probability.

### 3.3. AA Reduces Presynaptic Fiber Volleys and Postsynaptic Dendritic fEPSPs

**Figure 2:**
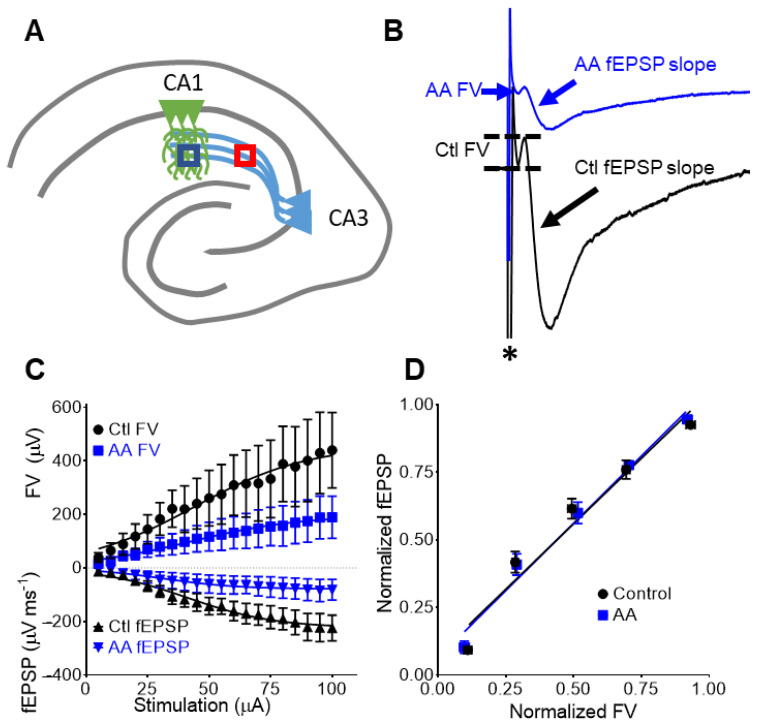
AA-mediated decrease in dendritic fEPSPs is due to diminished presynaptic fiber volleys (FV). (**A**) Schematic of the stimulation electrode (pink) in the CA3 schaffer collaterals and the recording electrode (blue) in the CA1 stratum radiatum. (**B**) Representative FV traces before (bottom black trace) and after AA application (top blue trace). An asterisk demarks the stimulation artifact. (**C**) AA effects on I/O curves for FV and fEPSPs obtained in the same recording (*n* = 4). (**D**) AA does not change FV-fEPSP coupling indicating the decreased fEPSPs results from the diminished FVs.

Next, the effect of AA on fiber volleys was assessed. Fiber volleys are generated by presynaptic axons of CA3 neurons in response to the stimulation as shown in Figure 2B. In recordings that contained visible fiber volleys, we found that 0.2 mM AA inhibited fiber volleys. The degree of inhibition was ~50%, similar to that of the dendritic CA1 fEPSPs (described in Section 3.1).

AA did not affect V_50_s of either fiber volleys or fEPSPs (Ctl FV = 44.2 ± 16.7 µA; AA FV = 42.2 ± 17.7 µA, *p* > 0.05; Ctl fEPSP = 41.4 ± 17.7 µA; AA fEPSP = 31.1 ± 11.1 µA, *p* > 0.05; paired *t*-test; *n* = 4) (Figure 2C). Fiber volley-fEPSP (FV–E) coupling ratios were obtained from the linear regression slopes of responses normalized to maximum values. Data were grouped according to normalized FV amplitude (bins of 0.2 units). FV-E plots revealed no difference in pre- and post-synaptic coupling (Figure 2D; regression slopes: 0.97 ± 0.12 vs. 1.0 ± 0.08; paired *t*-test; *p* = 0.86). These data indicate that the AA-mediated decrease in dendritic fEPSP slope is the result of diminished presynaptic fiber volleys.

### 3.4. In CA1, AA Augments Somatic fEPSP and Population Spike Coupling

**Figure 3:**
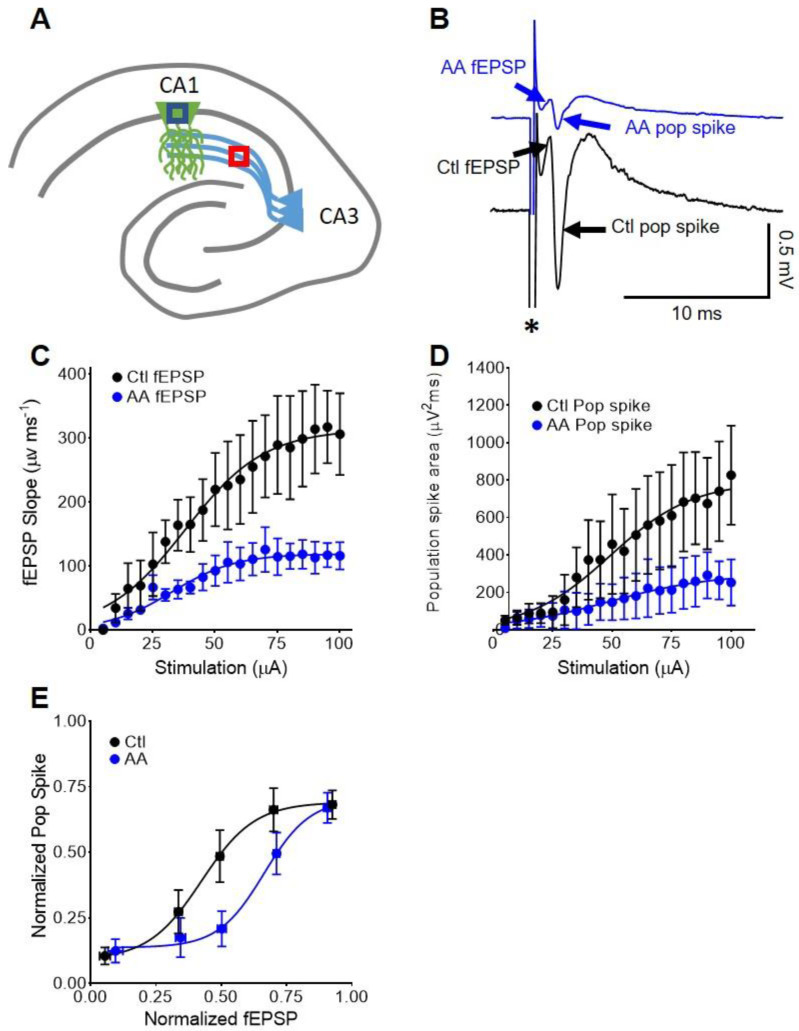
In CA1, AA Augments Somatic fEPSP and Population Spike Coupling. (**A**) Schematic of the stimulation electrode (pink) in the CA3 schaffer collaterals and the recording electrode (blue) in the CA1 stratum pyradmidale. (**B**) Representative traces of somatic fEPSPs and CA1 population spikes before (bottom black trace) and after AA application (top blue trace). An asterisk demarks the stimulation artifact. (**C**) Somatic fEPSP slope and (**D**) CA1 population spike area I/O demonstrate consistent AA-mediated inhibition throughout stimulation intensities (*n* = 4). (**E**) AA augments E-S coupling indicating intrinsic CA1 neuron excitability is attenuated.

To determine whether AA influenced the intrinsic excitability of the postsynaptic CA1 neurons, we assessed the coupling of excitatory inputs and firing efficiency. We analyzed electrodes in CA1 stratum pyramidale upon Schaffer Collateral stimulation (Figure 3A) and quantified the somatic fEPSP and the associated population spike (Figure 3B).

Compared to baseline, AA (2 mM) significantly reduced somatic CA1 fEPSPs (max. response: 116.2 ± 21.5 vs. 306.3 ± 63.8 µV ms^−1;^ paired *t*-test, *p* < 0.05; *n* = 4 slices; Figure 3C) and the population spikes (max. response: 252.9 ± 123.6 vs. 826.6 ± 264.7 µV*ms; paired *t*-test, *p* < 0.05; *n* = 4 slices; Figure 3D). The fEPSP-to-population spike (E–S) coupling was assessed by fitting normalized data with a non-linear regression and comparing the fEPSP needed to elicit a 50% population spike (E_50_). Data were grouped according to normalized fEPSP slope (bins of 0.2 units). E–S plots revealed a significant rightward shift of the E-S curve (E_50_: 0.42 ± 0.07 vs. 0.66 ± 0.06; paired *t*-test, *p* < 0.05; Figure 3E). These data indicate that in the presence of AA, a greater depolarization by somatic fEPSPs is required to generate a given magnitude of population spike, i.e., AA dampens the intrinsic excitability of CA1 neurons.

### 3.5. AA Reduces STP and LTP at the CA3-CA1 Synapse

**Figure 4:**
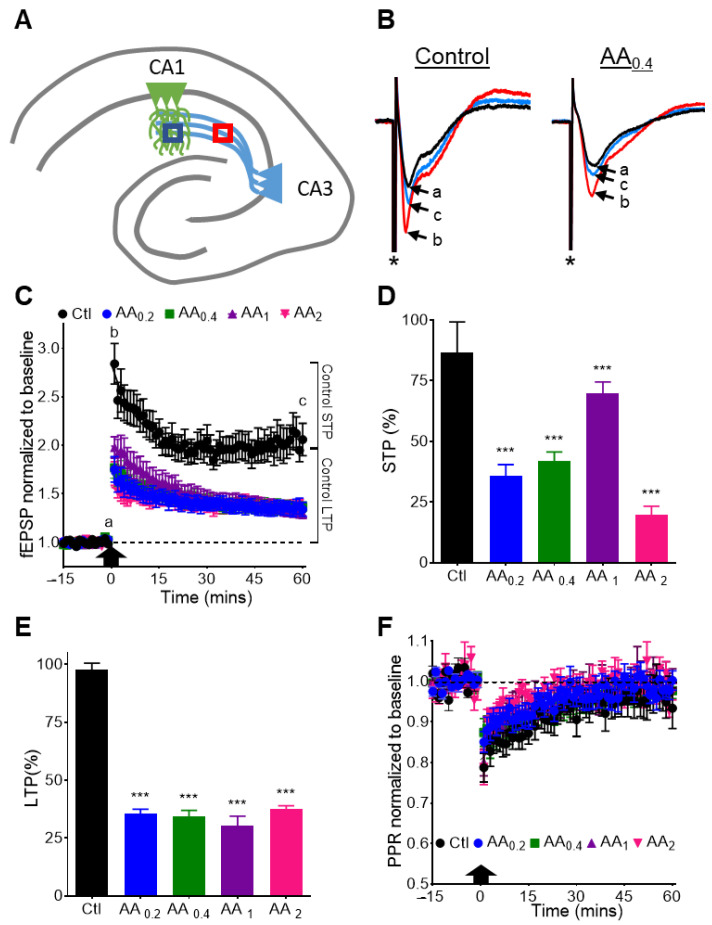
AA reduces STP and LTP. (**A**) Schematic of the stimulation electrode (pink) in the CA3 schaffer collaterals and the recording electrode(blue) in the CA1 stratum radiatum. (**B**) Representative traces of fEPSPs elicited by paired pulse stimulations before and after TBS from a control slice and a slice exposed to 0.4 mM AA. Trace overlays are relative to TBS: −1 min (black, a), +1 min (red, b) and +60 min (blue, c). Letters correspond to letters in panel B. Asterisks demark stimulation artifacts. Recordings were made in CA1 stratum radiatum. (**C**) Averaged fEPSP response to TBS (arrow). All concentrations of AA reduced STP and LTP. Data was fit with a mono-exponential equation and the span and plateau were obtained to calculate amplitude of potentiation for (**D**) STP and (**E**) LTP, respectively. (**F**) Normalized paired pulse ratios (PPR) indicate AA effects on STP and LTP were not due to alteration of neurotransmitter release mechanisms. Statistical analyses were performed with Welch’s ANOVA with Dunnett’s T3 multiple comparisons post-hoc test (*n* for Ctl = 4 mice, 4 slices, 19 electrodes; *n* for AA_0.2_ = 4 mice, 4 slices, 11 electrodes; *n* for AA_0.4_ = 4 mice, 4 slices, 14 electrodes; *n* for AA_1_ = 4 mice, 4 slices, 14 electrodes; *n* for AA_2_ = 4 mice, 4 slices, 11 electrodes). All values represent mean ± SEM; ***: *p* < 0.001.

To determine the impact of AA on other forms of plasticity we applied a TBS protocol to the Schaffer Collaterals and observed the effects on fEPSPs in the CA1 stratum radiatum dendritic region (Figure 4A,B). TBS elicits two forms of plasticity, a presynaptic-dependent STP lasting ~15 min and a postsynaptic-dependent LTP lasting hours to days. AA strongly inhibited TBS-induced STP (Figure 4C,D) and LTP (Figure 4C,E) in a concentration-independent manner. Paired pulse facilitation was decreased during STP demonstrating a presynaptic role for this form of plasticity (Figure 4F). AA did not affect this process suggesting AA-mediated reductions of STP and LTP does not involve alterations in presynatpic neurotransmitter release probabilities. These data suggest that AA exerts significant regulation of plasticity.

### 3.6. AA Reduces Spontaneous Sharp Waves and High Frequency Oscillations in Hippocampal Networks

**Figure 5:**
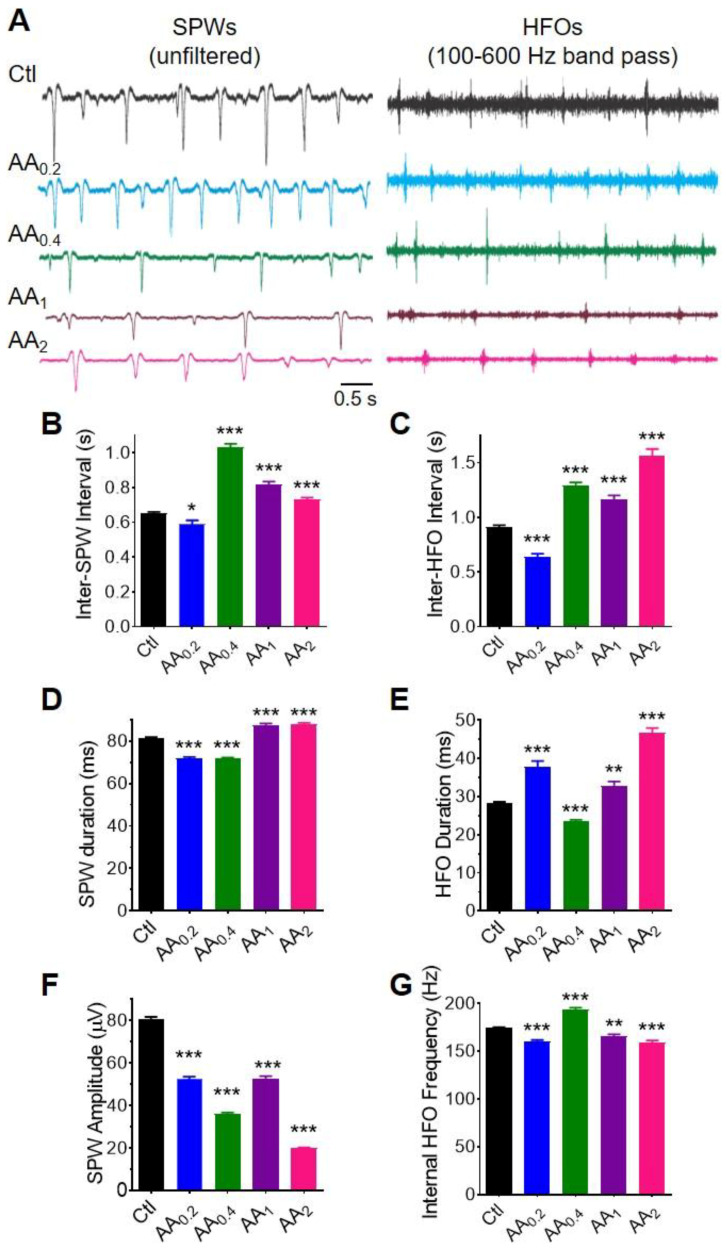
AA alters sharp waves (SPW) and high frequency oscillation (HFOs) architecture in the CA1 stratum radiatum. (**A**) Representative unfiltered recordings of spontaneous SPWs (left) from individual slices with or without AA and visualization of HFOs upon band pass filtering the same recordings (right). SPW-HFOs were recorded with electrode located in the CA1 stratum radiatum. Quantified characteristics of SPWs and HFOs include (**B**,**C**) interval, (**D**,**E**) duration, (**F**) HFO internal frequency and (**G**) SPW amplitude. AA affected characteristics with the general pattern of increasing the event interval (i.e., decreasing incidence), increasing duration, decreasing SPW amplitude and decreasing HFO internal frequency. Statistical analyses were performed with Welch’s ANOVA with Dunnett’s T3 multiple comparisons post-hoc test (*n* for Ctl = 16 mice, 16 slices; *n* for AA_0.2_ = 4 mice, 4 slices; *n* for AA_0.4_ = 4 mice, 4 slices; *n* for AA_1_ = 4 mice, 4 slices; *n* for AA_2_ = 4 mice, 4 slices). All values represent mean ± SEM. *: *p* < 0.05, **: *p* < 0.01, ***: *p* < 0.001.

To determine whether the effects of AA on evoked synaptic transmission and plasticity would impact hippocampal network activity, we examined the characteristics of sharp waves (SPWs) and associated high frequency oscillations (HFOs). In a healthy slice of ventral hippocampus, SPWs arise spontaneously in the CA3 region and propagate to CA1. SPWs are small field potentials that are 4–6x longer in duration than evoked fEPSPs and involve participation of a greater proportion of the region’s neurons. In CA1, a small number of the participating pyramidal cells and interneurons may fire actions potentials in synchrony giving rise to an oscillation or ‘ripple’ riding on top of the SPW usually in the frequency band of 100–250 Hz, an HFO (Figure 5A). During in vivo learning and memory tasks SPW-HFOs occur more frequently and have longer durations and SPW amplitude increases. Manipulation of these characteristics can improve or reduce performance on learning and memory tasks; therefore, we focused on these characteristics in assessing AA effects on SPW-HFOs.

AA had a bimodal effect on SPW and HFO occurrence (Figure 5B,C). For both SPWs and HFOs, 0.2 mM AA decreased interevent intervals (i.e., events happened 10% and 43% more often, respectively), whereas 0.4–2 mM AA increased interevent intervals (i.e., events happened 11–37% and 22–41% less often, respectively). Under control conditions 71% of SPWs had HFOs. The SPW-HFO association increased to nearly 1:1 with 0.2 mM AA (97%) and fell with higher concentrations to 80%, 70%, and 47% for 0.4, 1, and 2 mM, respectively.

AA also had a bimodal effect on SPW and HFO duration (Figure 5D,E). The lower concentrations of AA (0.2 and 0.4 mM) decreased SPW duration by 12%, whereas 1- and 2-mM AA increased duration by 8%. In contrast, HFO duration was increased by 34% with 0.2 mM, decreased by 17% with 0.4 mM and increased by 16% and 65% with 1 and 2 mM, respectively. AA did not affect the number of oscillations within a HFO (not shown); therefore, changes in duration were inversely reflected in the internal frequency of the HFO (Figure 5F). In contrast, the SPW amplitude was inhibited by AA in a concentration-dependent manner with an IC_50_ of 0.65 ± 0.18 mM and 80% inhibition by 2 mM (Figure 5G). Collectively, these data indicate that AA has profound effects on network activity.

## 4. Discussion

In the past 50 years, AA has emerged as a potent neuroprotectant and possible neuromodulator [4,8]. Often the neuroprotective properties of AA and their potential benefits in the neurobiology of disease and aging have overshadowed research into the neuromodulator role of AA, thus little is known. Here, we aimed to determine whether AA participates in neurotransmission, synaptic plasticity and neuronal network oscillatory activity at physiological concentrations in hippocampal slices from normal mice. We found: (1) AA reduces presynaptic fiber volley amplitude and fEPSP slope at CA3-CA1 synapses. (2) AA does not affect stimulation V_50_s, FV-E coupling or paired pulse facilitation (i.e., presynaptic neurotransmitter release probabilities); thus, AA reduces synaptic strength by attenuating presynaptic fiber volleys. (3) AA reduces CA1 neuronal E-S coupling indicating changes in intrinsic excitability. (4) AA reduces the magnitude of short- and long-term potentiation. (5) AA reduces the incidence of SPW-HFOs and reduces SPW amplitude.

There are few studies of direct effects of AA on neurotransmission or cellular excitability, but most suggest an inhibitory effect. In thalamic slices, Nelson et al. [12] found that AA (300 μM) reduced low-threshold calcium spikes in nucleus reticularis thalamic (nRT) neurons by 45%, preventing action potential bursting. Specifically, AA inhibited CaV3.2 T-type calcium channels [12]. Additionally, AA (500 μM) reduced evoked EPSPs by 78% in neocortical neurons and 3 mM AA enhanced GABA_A_ miniature inhibitory postsynaptic current (mIPSCs) amplitudes by 30% in bipolar cells of retinal slices [14,15]. Furthermore, greater enhancement of GABA currents occurred with low, ambient GABA concentrations found in extracellular fluid compared to high synaptic concentrations [15].

Acute in vitro brain slices lose 75–80% of endogenous tissue AA within the first 30 min of incubation [21]. Our experiments were performed on slices >2 h after the start of incubation; therefore, endogenous AA had minimal, if any, contribution to the effects we observed with application of exogenous AA. In line with the reports reviewed above, our results indicate that AA reduces the amplitude of presynaptic fiber volleys, dendritic and somatic fEPSPs and population spikes at the CA3-CA1 synapse in hippocampal slices. Inhibition of these parameters was not associated with alterations of synaptic function as there were no effects on facilitation, stimulation V_50_s, or FV-E coupling. AA did shift E-S coupling in CA1 so that greater depolarizations were necessary to fire action potentials, indicating AA decreases neuronal intrinsic excitability similar to the effect on nRT neurons [12]. Thus, AA dampens overall neuronal excitability.

How could this happen? Extracellularly recorded fiber volleys are the summation of action potentials occurring simultaneously in many axons (or fibers) in response to a stimulation. The fiber volley amplitude increases with increasing stimulation intensity and depends on the number of axons recruited by the stimulation, action potential amplitude and number of action potentials. The AA-mediated reduction in fiber volley amplitude could potentiallt be accomplished by decreasing the size of the stimulation-induced graded potential via regulation of low-threshold Cav3.2 T-type calcium channels or GABA_A_ receptors [12,15]. Further studies are needed to fully explore these mechanisms. Regardless, the decrease in fiber volley amplitude would result in decreased neurotransmitter release, smaller dendritic fEPSPs (but same FV-E coupling) and smaller CA1 somatic fEPSPs and population spikes.

Tetanic stimulation of presynaptic fibers induces various forms of plasticity at synapses including STP which involves enhancement of presynaptic neurotransmitter release probabilities and LTP which involves postsynaptic phosphorylation of and insertion of AMPA receptors (early stage) and protein synthesis (late stage), the result being an increase in synaptic strength for hours to months. We determined AA effects on STP and LTP and found that AA reduced both significantly. AA did not affect neurotransmitter release probabilities during STP as indicated by unchanged paired pulse facilitation dynamics suggesting a postsynaptic source for the reduction. At the CA3-CA1 synapse LTP is a graded response dependent on the strength of the stimulus [22,23]. Even though a ~V_50_ stimulation intensity was used in our LTP induction and AA did not affect the V_50_s, the amplitudes of the fiber volleys and consequent fEPSPs would be smaller in the presence of AA. This could reduce NMDA-receptor activation, calcium entry and protein kinase engagement resulting in lower STP and LTP. Alternatively, or additionally, AA could have direct effects on NMDA receptor function as it has been demonstrated to inhibit glutamate binding to NMDA receptors and decrease NMDA-currents in isolated neurons [24]. The effects on Cav3.2 channels and GABA_A_ receptors may play a role as well.

Brain waves or oscillations are emergent properties of neuronal network synchrony. Hippocampal networks generate a variety of oscillations including delta, beta, theta, and sharp waves as well as HFOs. Hippocampal oscillations occur spontaneously or in response to stimuli originating elsewhere in the CNS and participate in cognitive and memory processes. The highly laminar structure of the hippocampus preserves neuronal networks in acute slices in vitro providing experimental models for the study of these oscillations. A previous study found that AA (200 μM) resulted in a 30% reduction of the power and incidence of carbachol-induced theta wave bursts, oscillations important for planning and initiating movements and LTP [25]. Similarly, examining spontaneous SPW-HFOs, oscillations involved in memory consolidation, we found that AA reduces the amplitude of SPWs and incidence of SPWs and HFOs and increases the duration of SPWs and HFOs. Again, AA regulation of NMDA receptors, GABA_A_ receptors and Cav3.2 channels may mediate these effects as these receptor/channels have been demonstrated in initiating and shaping the characteristics of SPW-HFOs [26]. Specifically, NMDA receptor antagonism decreases SPW-HFO generation [27,28], GABA_A_ receptor agonists and allosteric modulators decrease SPW-HFO incidence, amplitude and increase duration [29,30] and T-type calcium channel blockers reduce the amplitude of SPWs [31]. AA is also involved in the synthesis, release, and uptake of monoamines and catecholamines, and α1-adrenergic receptors suppress SPW-HFOs, whereas β1-adrenergic receptors and dopamine 1/5 receptors promote SPW-HFOs [32,33]. Thus, the mechanism of action of AA on SPW-HFOs is likely complex and opposing effects may underlie the bimodal effects we observed for intervals and duration.

The role of ascorbic acid during homeostasis is thought to mostly involve its antioxidant, oxygen radical scavenging and enzymatic cofactor properties; therefore, its potential as a neuroprotective agent in neurodegenerative diseases, ischemia, stroke, and traumatic brain injury have been the primary focus of preclinical and clinical investigations [21,34]. The potential importance of a more direct and immediate role of AA as a neuromodulator is often overlooked even though AA has been demonstrated to regulate neurotransmitters, receptors and ion channels [12,14,15,24,25]. Collectively, these previous studies and our current study indicate that AA provides significant CNS inhibition and may have profound effects on neuronal plasticity, cognition, and memory. Traditionally, we would be prone to interpret these results as detrimental to CNS function [25], however, in this case the overwhelming evidence of AA beneficial effects suggests this interpretation is incorrect. Furthermore, the mere fact that AA is normally in brain tissue and CSF, and that most aCSF used for in vitro electrophysiological recordings lacks AA, suggests that most experiments study hyperexcitable tissue [35]. In this context the AA-mediated reduction of LTP, SPW-HFOs and theta waves would initially suggest a possible impairment of cognition and memory but is in fact reinstating more normal levels. In this light, by requiring stronger presynaptic activity AA may participate in refinement of signal processing and memory formation. Additionally, the AA-mediated dampening of hippocampal excitability that we found may participate in raising seizure thresholds. In fact, administration of AA to preclinical models of epilepsy reduces seizures [17,36,37]. In contrast, seizure threshold is reduced in a genetic mouse model of AA deficiency which is worsened when bred with a preclinical model of Alzheimer’s disease; and deficiency in dehydro-AA transport may contribute to seizures in GLUT1 syndrome [38,39,40]. This may be particularly important as AA levels are depleted or deficient in up to 30% of Western populations [41].

## 5. Conclusions

In conclusion, our results indicate that physiological concentrations of AA directly participate in neurotransmission and dampen excitability, plasticity, and network oscillations. Thus, AA may refine cognitive and memory processes and raise seizure thresholds, actively protecting the brain from pathologic excitability.

## Data Availability

The data that support the findings of this study are available from the corresponding author T.A.S. upon reasonable request.
